# 

**Published:** 1917-11-24

**Authors:** 


### CONTENTS.

PAGE

**The Homeland in War-Time** ... ... ... ... 153

**Mental and Muscular Work in Relation to Food** ... ... ... ... ... ... ... ... 154

**Hospital and Institutional News** ... ... ... 155

The British Hospitals Association—The Orthopædic Annexe—The Russian Red Cross—In Place of the Peace-time Dinner—The Growth of Local Laboratories—Generous Gifts—An Interesting Bequest—A Loss to Hampstead Hospital—An Inquiry Demanded at Lambeth Infirmary—Christmas Entertainments—First-Aid in Factories—Spotted Fever—"Our Day" in India—Unfounded Charges Against a Hospital—The Latest Scrap of Paper—The *Springbok Magazine*—The Scarcity of Sugar Substitutes—This Week's Drug Market—To Our Readers.

**Modern Artificial Limbs** ... ... ... ... ... 159

Their Influence upon Methods of Amputation.

**Decorations and Titular Honours** ... ... 161

Some New Developments and their Relation to the Hospital World.

**Village Practice** ... ... ... ... ... ... 162

An Autobiographical Fragment.

**The Food and Nutritional Diseases of an Army** ... ... ... ... ... ... ... ... 163

How the British Soldier is Cared For.

**Parliament and the Profession** ... ... ... 164

PAGE

**The Editor's Letter-Box** ... ... ... ... 165

The R.B.N.A. and the College.
Cleanliness in Public Buildings.

**Obituary** ... ... ... ... ... ... ... ... 165

**Nursing Affairs in Ireland** ... ... ... ... 166

The Midwives Bill.
Irish Nurses' League.

**The Matrons' and Sisters' Department** ... 167

The Nightingale School and St. Thomas's Hospital—III. (*Concluded.*)
Round the Hospitals.
Her Majesty's New Departure—A Memorable Event in British Nursing—The King and His Soldiers—What is Wrong with Glasgow?—Are Nurses' Rations Sufficient?

**School Attendance and Health** ... ... ... 171

**Commandeered Hospitals** ... ... ... ... 172

The Question of Compensation.

**Personalia** ... ... ... ... ... ... ... 172

**Matrons' and Sisters' Appointments** ... ... iv

**The Book Market** ... ... ... ... ... ... iv

**Gifts and Bequests** ... ... ... ... ... ... iv

**The Institutional Worker** ... ... (Suppl.) 1

An Isolation Hospital Difficulty.
What to Write in a Visitors' Book.

